# Effects of pain associated with orthodontic tooth movement on tactile sensation of periodontal ligaments

**DOI:** 10.1007/s00784-023-05469-2

**Published:** 2023-12-26

**Authors:** Eriya Shimada, Hiroyasu Kanetaka, Hiroki Hihara, Akitake Kanno, Ryuta Kawashima, Nobukazu Nakasato, Kaoru Igarashi

**Affiliations:** 1 Division of Craniofacial Anomalies, Tohoku University Graduate School of Dentistry, Sendai, Japan; 2 Department of Orthodontics and Speech Therapy for Craniofacial Anomalies, Tohoku University Hospital, Sendai, Japan; 3 Division of Interdisciplinary Integration, Liaison Center for Innovative Dentistry, Tohoku University Graduate School of Dentistry, Sendai, Japan; 4 Division of Advanced Prosthetic Dentistry, Tohoku University Graduate School of Dentistry, Sendai, Japan; 5 Department of Advanced Spintronics Medical Engineering, Tohoku University Graduate School of Engineering, Sendai, Japan; 6 Department of Functional Brain Imaging, Institute of Development, Aging and Cancer, Tohoku University, Sendai, Japan; 7 Department of Epileptology, Tohoku University Graduate School of Medicine, Sendai, Japan

**Keywords:** Magnetoencephalography, Orthodontic tooth movement, Pain, Somatosensory evoked magnetic fields, Tactile stimulation

## Abstract

**Objectives:**

Pain associated with orthodontic tooth movement reportedly reduces periodontal ligament tactile sensation. However, the mechanism associated with the central nervous system remains unclear. This study was conducted by measuring somatosensory evoked magnetic fields (SEFs) during mechanical stimulation of teeth as they were being moved by separator elastics. Findings clarified the effects of pain on periodontal ligament tactile sensation during orthodontic tooth movement.

**Materials and Methods:**

Using magnetoencephalography, SEFs were measured during the application of mechanical stimuli to the mandibular right first molars of 23 right-handed healthy participants (0 h). Separator elastics were subsequently inserted into the mesial and distal interdental portions of the mandibular right first molars. The same mechanical stimuli were applied again 24 h later while the SEFs were measured (24 h). After each SEF measurements, pain was also evaluated using the Visual Analog Scale (VAS).

**Results:**

The VAS values were significantly higher at 24 h than at 0 h (*p* < 0.05). No significant difference in the peak latencies was found between those obtained at 0 h and 24 h, but the intensities around 40.0 ms in the contralateral hemisphere were significantly lower at 24 h than at 0 h (*p* < 0.01).

**Conclusions:**

Pain associated with orthodontic tooth movement might suppress periodontal ligament tactile sensation in the primary somatosensory cortex.

**Clinical Relevance:**

Pain associated with orthodontic tooth movement might affect periodontal ligament sensation, consequently causing discomfort during occlusion.

## Introduction

When the periodontal ligament responds to loads applied to the tooth, it produces tactile, pressure, and vibration sensations [1–3]. Periodontal ligament sensations are extremely sensitive, able to detect even micrometer thickness [4, 5] and playing important roles in detecting food mass size and hardness [6] and in adjusting the jaw position and bite force [7]. The presence of the periodontal ligament is also necessary for moving teeth. When orthodontic forces are applied to the teeth, cytokines such as IL-1, IL-6, TNF-α, INF-γ, and M-CSF are released from the compression side of periodontal ligament, stimulating monocyte-derived macrophages and osteoclasts to promote alveolar bone remodeling [8–11]. This remodeling causes the tooth to move forward on the side of the compression. As the orthodontic force moves the tooth, prostaglandins are released. They subsequently bind to the sensory nerve endings in the periodontal ligament, resulting in pain. The nerve fibers innervating the periodontal ligament receptors are the superior alveolar nerve in the maxilla and the inferior alveolar nerve in the mandible, which comprise Aβ, Aδ, and C fibers [12]. Actually, Aβ fibers transmit tactile sensations, with a 65.0–70.0 m/s transmission speed, whereas Aδ and C fibers transmit pain and temperature, with Aδ fibers transmitting at 10.0–15.0 m/s and 30.0 m/s at the earliest [13, 14]. Action potentials generated in peripheral nerves by pain stimulation pass through the dorsal horn of the spinal cord and then the thalamus. They are input to the cortical somatosensory cortex. In addition, this action potential can enter the limbic system (amygdala, insula, anterior cingulate gyrus, etc.) via the thalamus. Alternatively, it can enter the limbic system via the brainstem without passing through the thalamus. The somatosensory cortex recognizes sensory aspects of pain such as the intensity, site of stimulation, and duration, whereas the limbic system is reported to recognize emotional and cognitive aspects of pain such as discomfort. Information from these two directions is integrated in the prefrontal cortex [15]. Such pain reaches a peak after one day of orthodontic force loading, eventually disappearing after 3–7 days [16].

In recent years, it has become clear that interaction exists between human pain and tactile sensations. An earlier study for which acute lower back pain was produced temporarily and the two-point discrimination zone of the lower back pain site was assessed found tactile acuity to be significantly worse than before the pain [17]. Several other reports have described that tactile acuity is dulled by pain in various parts of the body [18–21]. In fact, regarding oral sensation, when capsaicin was applied to the gingiva to induce pain, significantly lower sensitivity to mechanical stimulation was found than before capsaicin application [22]. A pain-inducing study in which capsaicin was injected into the periodontal ligament showed the mechanical detection threshold of the adjacent gingiva as significantly higher [23]. Bucci et al. evaluated changes in occlusal contact sensitivity caused by pain associated with orthodontic tooth movement, particularly pain induced by wearing separator elastics. The separator elastics were removed after being worn 24 h in the mesial and distal interproximal maxillary first molars. Occlusal tactile acuity was evaluated by having the teeth bite aluminum foil of various thicknesses. The results demonstrated that occlusal tactile acuity was significantly lower than that before the use of separator elastics [5]. Stimulation of brain regions associated with the pain response presumably interferes with and delays the transmission of tactile stimulation, possibly reducing tactile sensation. However, for that earlier study, only subjective evaluations were used to make measurements. Moreover, the associated mechanism remains unclear.

Therefore, this study specifically applied magnetoencephalography (MEG), a noninvasive functional brain imaging measurement, which measures the magnetic field around electrical signals generated in the brain. Unlike electroencephalography, MEG is less likely to record components deeper than the cortex, making it easier to record cortical components selectively [24]. For the study described herein, MEG is a very suitable device for objectively evaluating changes in periodontal ligament tactile sensation caused by pain by recording signals in the primary somatosensory cortex. Although reports have described periodontal ligament tactile sensation evaluated using MEG [25, 26], no report has described neuroscientific evaluation of the periodontal ligament tactile sensation on teeth in which pain results from orthodontic forces.

The working hypothesis examined for this study is that pain caused by orthodontic tooth movement decreases the signal intensity recorded in the primary somatosensory cortex in response to periodontal ligament tactile stimulation. This study, which uses MEG to measure somatosensory evoked magnetic fields (SEFs) by periodontal ligament tactile stimulation, was designed to elucidate the effects of pain induced by orthodontic tooth movement on periodontal ligament tactile sensation.

## Participants and methods

### Participants

Of the initial 33 persons approached to participate in the study, 23 healthy volunteers (7 women, 16 men, mean age 23.3 years) participated in the study. Inclusion criteria were (1) 18–35 years old and (2) right-handed. The Edinburgh handedness test was used for evaluation to confirm the handedness of each. Exclusion criteria were (1) undergoing orthodontic treatment, (2) having restoration covering the cusp of the mandibular right first molar such as an inlays or crown, or (3) having a history of neurological disease. This study was approved by the Ethics Committee of the Tohoku University Graduate School of Dentistry (protocol number: 26–39). Informed consent was obtained from all participants. Parental consent was obtained for participants who were minors.

### Stimulation

**Fig. 1:**
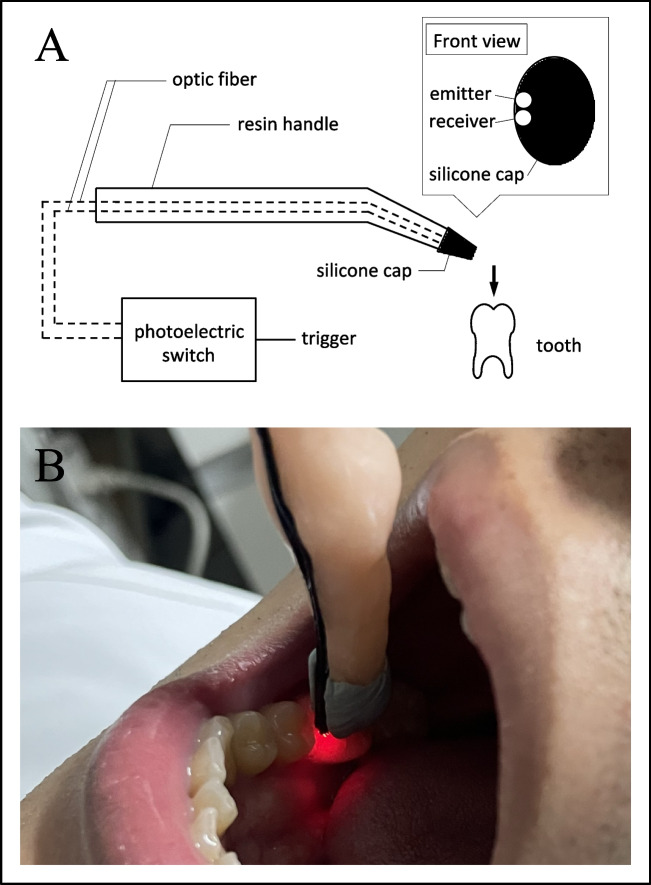
(**A**) Schematic diagram of the periodontal ligament stimulator, which consists of a resin handle and a silicone cap. At the tip of the silicone cap, an optical fiber emitter and receiver are installed. The optoelectronic switch detects the moment when the stimulator contacts the tooth. The red light is blocked. Then the reflected light is no longer perceived as a trigger (referred from Shimada et al., 2022). (**B**) Actual condition of a mandibular right first molar stimulated using the periodontal ligament stimulator

Mechanical stimulation was performed on the mandibular right first molar and left wrist using a homemade stimulator based on the brush stimulator described by Jousmäki et al. [27]. The stimulator consists of a resin handle, a silicone cap, and two optic fibers (E32-DC200F4R; Omron Corp., Kyoto, Japan) (Fig. 1) [26]. The fiber is attached along the resin handle. The fiber tip is in the silicone cap attached to the end of the resin handle. Mechanical stimulation was applied by lightly tapping the occlusal surface of the teeth with the silicone cap part. The optic fibers are connected to a photoelectric switch (E3X-NA41F 2 M; Omron Corp.): one fiber emits red light; the other senses the reflected light. The photoelectric switch recognizes the moment at which the silicone cap contacts the tooth surface. The reflected light is no longer detected as the trigger point.

To measure baseline cortical responses, mechanical stimuli were applied 300 times each to the mandibular right first molar and left wrist (0 h). The stimulus intensity was approximately 100 g. The stimulation interval was 0.5–1.0 s. All mechanical stimuli were applied by a single trained dentist. Therefore, constant force was regarded as having been applied. After mechanical stimulation, separator elastics were placed on the mesial and distal interproximal mandibular right first molar for 24 h. The separator elastics were removed 24 h later. Mechanical stimulation was applied again (24 h). As a subjective evaluation method, the Visual Analog Scale (VAS) [28] was used to assess pain immediately after mechanical stimulation: at 0 h and 24 h. The results were compared along with SEFs.

### MEG recordings

MEG data were recorded in a magnetically shielded room using a whole-head 200-channel MEG system (PQA160C; Ricoh Co., Ltd., Tokyo, Japan). The head morphology of each subject was digitized using a three-dimensional digitizer (Fast SCAN Cobra; Polhemus Inc., Colchester, VT). Individual structural magnetic resonance images obtained using a 3 T MR system (Achieva; Philips Healthcare, Best, the Netherlands) were co-registered. After the MEG signals were recorded from 50.0 ms before stimulation to 300.0 ms after the trigger point, they were bandpass filtered from 20 to 500 Hz, and were digitized at 1000 Hz.

### Data analysis

The signal sources and moments corresponding to the peak latency were evaluated individually using the signal equivalent current dipole (ECD) model calculated using analysis software (MEG Laboratory; Ricoh Co., Ltd.). The ECD model, based on Sarvas law [29], assumes a spherical conductor to identify the magnetic signal source. The signal source location was evaluated separately in the left and right hemispheres. Bilateral hemispheres were analyzed for this study. The resulting data were averaged for 300 stimulation data after removing visually obvious noise. Baseline levels were set at 4.0–9.0 ms after mechanical stimulation. Shimada et al. reported a MEG study showing first waves in the primary somatosensory cortex at 41.7 ms ± 5.70 ms after mechanical stimulation of the mandibular first molar periodontal ligament [26]. In addition, Umino et al. reported a study using EEG, which showed correlation between the intensity of electrical stimulation of the teeth and the slow component of 150–300 ms [30]. Therefore, for this study, responses with a signal source in the central sulcus in the first wave (early component) were observed at around 40.0 ms and in the second wave (late component) observed between 70 and 300 ms were the target of evaluation. Dipole locations were superimposed on MR images; ECDs were in the central sulcus. Only responses with goodness-of-fit values greater than 80% were selected. For statistical analyses, unpaired *t*-tests were used for peak latency and intensity between 0 and 24 h. Paired* t*-tests were used for VAS.

## Results

In the primary somatosensory cortex, the early component represents periodontal ligament tactile sensation. The late component represents periodontal ligament pain sensation. The number of early contralateral hemisphere components detected during mechanical stimulation of the mandibular right first molar at 0 h and 24 h were 11/23 (0 h) and 9/23 (24 h). Those for the ipsilateral hemisphere early components were 4/23 (0 h) and 5/23 (24 h). Those for the contralateral hemisphere late components were 7/23 (0 h) and 3/23 (24 h). In addition, those for the ipsilateral hemisphere late component were 7/23 (0 h) and 2/23 (24 h). The detection rate of the contralateral hemisphere during left wrist stimulation was 22/23. The 24 h detections of the ipsilateral hemisphere late component were so few (2 cases) that no test of significance was possible.

**Fig. 2:**
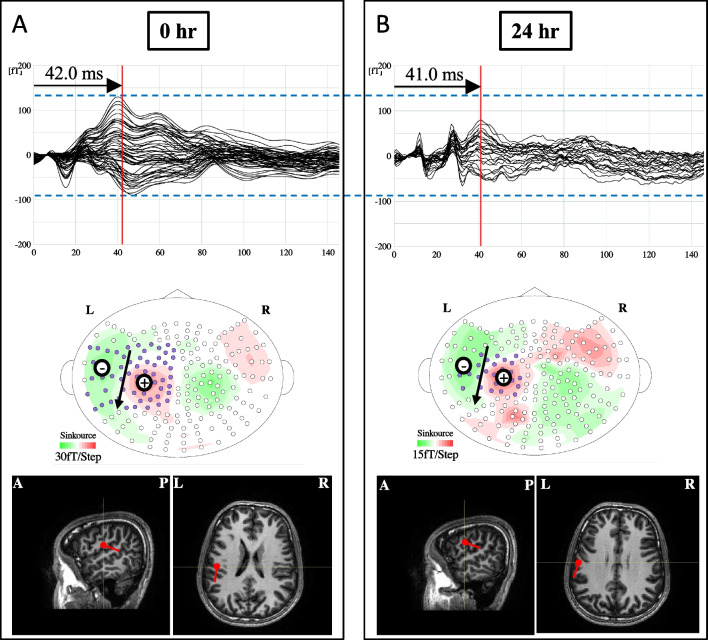
Somatosensory evoked magnetic fields in the contralateral hemisphere during mechanical stimulation of the mandibular right first molar of a 20-year-old male participant. The whole-head magnetic waveforms, isofield maps, and ECD locations from the upper panels: (**A**) 0 h and (**B**) 24 h. The amplitude of the waveform around 40.0 ms at 24 hr is smaller than that at 0 hr

**Fig. 3:**
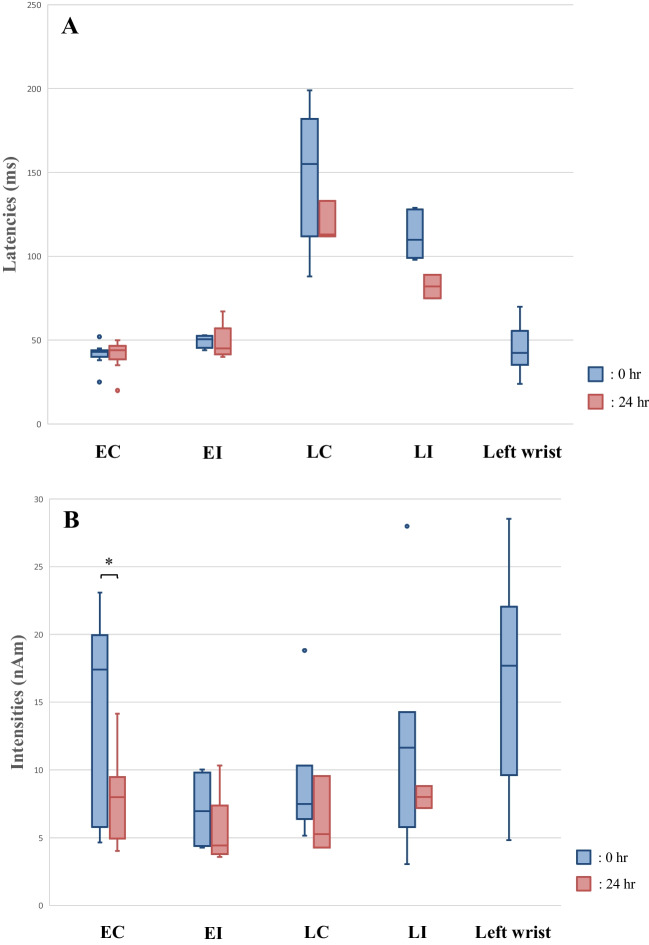
(**A**) Latencies (ms) and (**B**) intensities (nAm) for the mandibular right first molars and left wrists: EC, early component of contralateral hemisphere; EI, early component of ipsilateral hemisphere; LC, late component of contralateral hemisphere; LI, late component of ipsilateral hemisphere. No significant difference was found between the latencies of 0 h and 24 h for the respective components. The intensities of the waveforms observed in the contralateral hemisphere at 40.0 ms (early components) were significantly lower at 24 h than at 0 h (*p < 0.01)

The peak latency represents the time from periodontal ligament stimulation until the signal is transmitted to the primary somatosensory cortex. The peak latencies (mean ± SD) during mechanical stimulation of the mandibular right first molar were 41.5 ± 6.2 ms (0 h) and 41.3 ± 8.5 ms (24 h) for the contralateral hemisphere early component, 49.5 ± 3.4 ms (0 h) and 48.4 ± 9.6 ms (24 h) for the ipsilateral hemisphere early component, 151.6 ± 36.9 ms (0 h) and 119.3 ± 9.7 ms (24 h) for the contralateral hemisphere late component, 113.4 ± 12.9 ms (0 h), 82.0 ± 7.0 ms (24 h) for the ipsilateral hemisphere late component, and 45.6 ± 13.1 ms during mechanical stimulation at the left wrist. No significant difference was found between them (Figs. 2 and 3A).

Reportedly, the intensities of the waveforms around 60.0 ms observed in the contralateral hemisphere primary somatosensory cortex correlate with the tactile stimulation strength sensed in the periphery [31, 32]. Primary somatosensory cortex responses around 150.0–300.0 ms correlate with pain intensity [30, 33]. The signal intensity during mechanical stimulation of the mandibular right first molar was 14.0 ± 6.7 nAm (0 h) and 7.9 ± 3.0 nAm (24 h) for the early component of the contralateral hemisphere, 7.1 ± 2.6 nAm (0 h) and 5.4 ± 2.5 nAm (24 h) for the early component of the ipsilateral hemisphere, 9.1 ± 4.2 nAm (0 h) and 6.4 ± 2.3 nAm (24 h) for the late component of the contralateral hemisphere, 11.8 ± 7.6 nAm (0 h) and 8.0 ± 0.8 nAm (24 h) for the ipsilateral hemisphere late component, and 16.5 ± 6.5 nAm during mechanical left wrist stimulation. In the contralateral hemisphere early component, the signal intensity was significantly lower at 24 h than at 0 h (*p* < 0.05) (Figs. 2 and 3B). Otherwise, no significant difference was found.

**Fig. 4:**
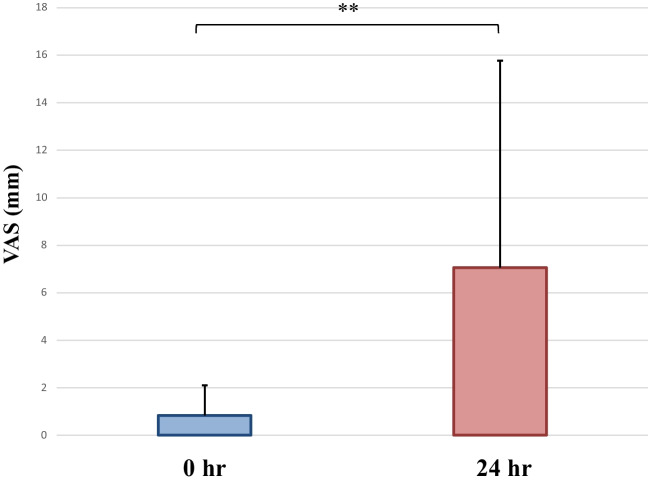
VAS (mm) values during mechanical stimulation of mandibular right first molars. The value for 24 h was significantly greater than that for 0 h (**p < 0.05)

The VAS during mechanical stimulation of the mandibular right first molar was 0.83 ± 1.27 mm (0 h) and 7.06 ± 8.71 mm (24 h). A significant greater VAS was found at 24 h than at 0 h (*p* < 0.05, Fig. 4).

## Discussion

This report is the first of a study examining the pain effects produced by orthodontic tooth movement on periodontal ligament tactile sensation. No difference in the peak latency of the bilateral hemispheres was observed between 0 and 24 h. The intensities in the early components of the contralateral hemispheres were significantly lower at 24 h than at 0 h. The VAS evaluation found a significantly greater subjective degree of pain at 24 h than at 0 h.

### Peak latency

Considering transmission rates, the waveforms observed around 41.0 ms, which are the early components, are apparently responses by Aβ fibers, whereas the waveforms observed between 150.0 and 300.0 ms are regarded as a response by Aδ fibers [33]. Results of this study suggest that both Aβ and Aδ fibers are stimulated by mechanical stimulation of the periodontal ligament, but in neither hemisphere was a significant difference in peak latency found at 24 h compared to 0 h. Tran et al. reported a study using MEG indicating that the presence or absence of hand pain stimulation did not affect the peak latency in the response around 30.0 ms detected in the primary somatosensory cortex with non-painful stimulation [34]. This lack of effect was attributable to saturation of the activated Aβ fibers in both non-painful and painful stimulation sessions. In the present study, mechanical stimulation of 100 g was also applied to the periodontal ligament at 0 h and 24 h. Because all Aβ fibers were activated in both sessions, it is considered that there were no changes in peak latencies with or without pain associated with orthodontic tooth movement.

### Comparison of VAS and intensity

Interleukin 1-beta, which regulates bone remodeling, is produced when orthodontic forces are applied to the teeth, but such forces also induce the secretion of inflammatory mediators, which correlates with the intensity of pain produced during orthodontic treatment. Interleukin 1-beta increases most during 24 h after orthodontic forces are applied to the teeth [35]. Moreover, pain associated with orthodontic tooth movement develops about 2 h after tooth movement begins, peaking approximately 24–48 h later, and subsiding gradually thereafter [36]. For the evaluation of VAS in this study, as explained also in earlier reports, the degree of pain was significantly greater 24 h after the elastics were applied.

The intensities were significantly lower at 24 h, even though the same strength of periodontal ligament mechanical stimulation was applied at both 0 h and 24 h. The results presented herein indicate that orthodontic tooth pain reduces periodontal ligament tactile sensation. Several reports have described studies of pain increasing tactile thresholds. In fact, after capsaicin was injected into the forearm, tactile thresholds were found to be significantly higher in the hyperalgesic region than in the control region when evaluated using von Frey hairs. The mechanism is that primary afferent depolarization induced by C fibers cause presynaptic inhibition of low-threshold mechanoreceptors, which leads to reduced tactile sensitivity [37]. When acute peripheral nociceptive back pain was induced and the two-point discrimination zone was measured, tactile sensitivity was markedly reduced in the back pain-induced group compared to the control group. That study concluded that, although all subjects in the back pain-induced group received the same amount of saline solution in the lower back, their subjective pain experiences varied. The peripheral nociceptor mechanism was not the only mechanism causing changes in tactile sensation [17]. For this study, constant mechanical stimulation was also applied, but large variation was found in the perception of tooth pain, as assessed using the VAS. The pain was markedly more intense after insertion of the separator elastics, but the individual participants’ perceptions of the pain varied greatly. Therefore, it is still not possible to explain this mechanism based solely on peripheral nociceptors.

Bucci et al. studied how pain and periodontal ligament stretching caused by tooth movement affect periodontal ligament tactile sensation [5]. Occlusal tactile acuity during occlusion was measured before insertion of the separator elastics, 24 h after insertion of the elastics between the mesial and distal maxillary first molars, and 7 days later. The results demonstrated that the tactile thresholds of occlusion of 24-μm and 32-μm-thick aluminum foil were significantly higher at 24 h after insertion of the separator elastics, when the pain peak was reached, than they were before insertion of the separator elastics. Results demonstrated that the tactile sensation of the periodontal ligament is dulled by pain caused by orthodontic tooth movement and by pain caused in other parts of the body. This phenomenon has been regarded as involving a "touch gate" [38] that works similarly to the gate control theory of pain [39]: pain diminishes the tactile sensation. Tran et al. reported, when non-painful stimulation was applied to the wrist, that the waveform observed at 30.0 ms in the primary somatosensory cortex originated in area 3b. In humans, pain information transmitted through Aδ fibers reaches primary somatosensory cortex 1, suggesting that the inhibition of tactile sensation results from inhibitory interneurons connecting cortex 1 and 3b [34]. Osaki et al. used mice to examine how painful and tactile information is processed in the primary somatosensory cortex [40]. The results demonstrated that layer 5 neurons in the dysgranular region (the region which mainly processes pain sensation) were inactivated by tactile stimulation, whereas the neurons of layer 2/3 in the barrel region (the region that processes touch sensation), were inactivated by nociceptive stimulation. Periodontal ligament tactile sensation might also interact in the primary somatosensory cortex with pain caused by orthodontic tooth movement, leading to the suppression of tactile sensation. The phenomenon of suppression of periodontal ligament tactile sensation by pain associated with orthodontic tooth movement is likely to involve the primary somatosensory cortex, but details of the related mechanisms require further investigation.

Discomfort associated with eating during orthodontic treatment is often associated with the foreign body sensation of the appliance or pain during biting [41, 42]. Moreover, in addition to these factors, tooth hypersensitivity might be a contributing factor. Using MEG for this study, we objectively evaluated the effects of pain associated with orthodontic tooth movement on periodontal ligament tactile sensation, which provided us insight into the mechanism of interaction between pain and tactile sensation in the human central nervous system that had not been elucidated in earlier studies. These findings are expected to lead to future insights into the development of new orthodontic treatment methods able to reduce or eliminate the oral discomfort which invariably occurs during orthodontic treatment.

## Conclusion

Results of this study suggest that pain associated with orthodontic tooth movement suppresses periodontal ligament tactile sensation in the central nervous system. Moreover, SEFs were demonstrated to be useful for the objective assessment of periodontal ligament tactile sensation.
